# Biostimulants in plant brassinosteroid hormone receptor BRI1 activation—a new system to evaluate activation capacity

**DOI:** 10.1111/febs.70235

**Published:** 2025-08-28

**Authors:** Maribel Marquina, Montserrat Ricart‐Fort, Rocío Díaz‐Parra, Sandra López‐Avilés, Tula Yance, Pablo Quirós, Elena Contreras, Ignasi Salaet, Sergio Atares, Rosa Aligué

**Affiliations:** ^1^ Department of Biomedical Science University of Barcelona Spain; ^2^ Biosanitary Research Institute Valencian International University (VIU) Valencia Spain; ^3^ Department of Biosciences, Faculty of Mathematics and Natural Sciences University of Oslo Norway; ^4^ Departamento de I+D+i de Fertinagro Biotech S.L Poligono Industrial La Paz Teruel Spain

**Keywords:** bioactive natural products, biostimulants, brassinolide, brassinosteroids receptors, fission yeast, sustainable agriculture

## Abstract

The search for innovative and alternative chemical methods to manage plant growth is an ever‐increasing reality. Biostimulants, products of biological origin, have shown promise in improving various agronomic characteristics and boosting yield. However, the selection and characterization of biostimulant matrices is a complex process that requires rigorous evaluation adapted to the specific needs of each plant. Because mixtures of biologically active compounds are present in biostimulants, efficient methods are required to characterize their potential mode of action. In this study, a new approach was developed to assess the biological activity of biostimulants by activating specific plant receptors involved in key physiological processes. It is based on the heterologous expression in fission yeast of brassinosteroid receptor protein Brassinosteroid Insensitive 1 (BRI1), which is involved in plant growth and development, and its specific activation by brassinolide (BL). The method involves the identification of highly expressed genes in response to BL activation of the BRI1 receptor, to generate a GFP reporter gene system that is switched on when biostimulants activate the BRI1 receptor. The biostimulants selected for testing were hydrolysates of animal origin. The results not only revealed variations in BRI1 activation among biostimulants, but also highlighted that samples from the same origin exhibit different BRI1 activation capacities depending on their processing methods. This new method enables direct classification of the mode of action of biostimulants by assessing their ability to activate specific plant receptors, providing a valuable resource for biostimulant research and development.

## Graphical abstract

Biostimulants improve plant growth and have shown promise in agronomic applications and boosting crop yield. Because biostimulants typically present as a mixture of active components, new methods are required to characterize their mode of action. Here, we describe a novel assay that evaluates plant biostimulants targeting the brassinosteroid receptor BRI1, a central component in plant growth and development. Expressing BRI1 in fission yeast enabled its functional activation by brassinolide (BL) and downstream transcriptomic profiling identified BL‐responsive genes via a GFP‐based reporter system. This platform offers a robust, specific, and high‐throughput method to screen new biostimulants that influence plant growth through BRI1 signalling.

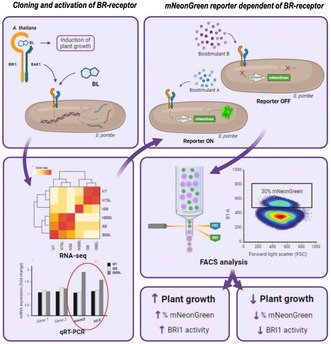

**Abbreviations**
BAK1: brassinosteroid‐insensitive receptor‐associated kinase 1
BL: brassinolide
BRI1: brassinosteroid insensitive 1
BRs: brassinosteroids
CCiTUB: cytometry unit from scientific and technological centres
DEGs: differentially expressed genes
EMM2: Edinburgh minimal medium 2
GFP: green fluorescent protein
GO: gene ontology
IC50: inhibitory concentration 50
KEGG: Kyoto Encyclopaedia of Genes and Genomes
MAPK: mitogen‐activated protein kinase
OD: optical density
ORF: open reading frame
PC: principal component
RIN: RNA integrity number
*S. cerevisiae*
: *Saccharomyces cerevisiae*
*S. pombe*
: *Schizosaccharomyces pombe*

## Introduction

The biostimulants industry has seen significant expansion in recent times. Numerous companies are actively developing and launching novel products and components [1, 2, 3, 4]. The sources of biostimulants are diverse, encompassing bacteria, fungi, algae, higher plants and animals, and they range from individual compounds to intricate mixtures with partially identified bioactive elements [5, 6, 7]. Despite their complexity, biostimulants contain a wide array of plant signalling molecules, such as plant hormones, amino acids and polyamines [7, 8].

Knowledge about the benefits of biostimulants in plants is constantly improving. The composition and development of biostimulants is being studied utilizing a wide range of methodological approaches, such as chemical and non‐chemical composition characterization [5, 9, 10], plant growth and yield studies [11, 12, 13] and genomic, proteomic and metabolomic analyses [14, 15, 16, 17]. Understanding the mechanism of action is equally important. Integrating data from chemistry, biology and omics helps evaluate the activities and synergies of natural compounds and microorganisms used in agriculture [13, 18]. Furthermore, the biological activity of plant biostimulants has also been tested using a high‐throughput screening of multiple traits of *Arabidopsis* characteristics to define its contribution to plant development and stress tolerance [19, 20].

This work proposes a novel approach to characterize the mode of action of biostimulants in regulating plant growth and development based on the capacity of its components to activate specifically the brassinosteroid steroid hormone receptor class, BRI1, expressed in yeast.

Brassinosteroids (BRs) are a group of plant steroid hormones involved in many aspects of plant growth and development [21, 22, 23, 24]. BR hormones are sensed extracellularly by membrane‐bound members of the leucine‐rich repeat receptor‐like kinase [25, 26], such as Brassinosteroid Insensitive 1 (BRI1). BR hormone binds directly to the extracellular domain of BRI1 [27, 28, 29, 30] and triggers the formation of the Brassinosteroid‐Insensitive Receptor‐Associated Kinase 1 (BRI1‐BAK1) heterodimer, which in turn initiates an intracellular phosphorylation cascade leading to transcription of BR‐responsive genes that drive cell growth [31, 32, 33, 34].

On the contrary, it is not yet known whether the BRI1 receptor can be activated by other molecules or components of biostimulants. To evaluate the new approach, biostimulants derived from animal protein hydrolysates resulting from partial hydrolysis and primarily consisting of signalling peptides and free amino acids were utilized [11, 35]. The composition of protein hydrolysates is determined by the source of protein (animal or plant) and the method of hydrolysis employed (chemical, thermal and/or enzymatic) [36, 37]. Protein hydrolysates have gained popularity as a non‐microbial biostimulant due to their capacity to enhance plant growth, yield and overall quality [11, 36, 38, 39]. In sustainable agriculture, animal protein hydrolysates have shown promising results in promoting plant growth and mitigating environmental stresses [37, 40]. They enhance crop physiology by reinforcing natural plant defences and improving nitrogen utilization efficiency through the activation of N metabolism enzymes [11, 40, 41]. Moreover, their use as a biostimulant is considered cost‐effective, as most raw materials are derived from animal waste [11, 40, 41]. To develop the new system, the fission yeast *S. pombe* was selected as a cellular model. Yeast has already been used as a model system to evaluate biostimulants [13]. Yeast cells, including *S. cerevisiae* and *S. pombe*, provide an amenable system for the heterologous expression of genes. Yeast cells have a fast generation time and are inexpensive to cultivate. In addition, unlike prokaryotic organisms, they are rich in ER and mitochondrial membranes and can carry out post‐translational modifications that facilitate the folding of foreign proteins [42]. Among the commonly used yeasts, *S. pombe* stands out due to its advanced membrane structure, which is particularly advantageous for recombinant expression of membrane proteins [43]. Furthermore, fission yeast is a prominent model organism for unravelling the molecular mechanisms underlying fundamental cellular processes [44]. Overall, *S. pombe* is an excellent model system for heterologous protein expression and compound screening.

This study introduces a novel approach for assessing biostimulant biological activities. The method utilizes *S. pombe* as a model organism for heterologous expression of the BRI1 brassinosteroid receptor, which plays a crucial role in plant growth and development. Through specific activation of the BRI1 receptor using brassinolide (BL), highly expressed genes in response to BL activation were identified. The most upregulated genes were then selected to create a reporter gene‐GFP system, which becomes activated when biostimulants trigger the BRI1 receptor. The new method aims to establish the biological properties of biostimulants, in this case, and their ability to activate a receptor involved in plant growth and development, providing an easy test to evaluate multiple biostimulants.

The approach, when applied to biostimulants used in this work, identifies which ones can trigger the BR1 receptor and to what extent. This demonstrates that the method is both qualitative and quantitative in nature.

## Results

### Conditional expression and membrane localization of BAK1 and BRI1 plant receptors in fission yeast

**Fig. 1:**
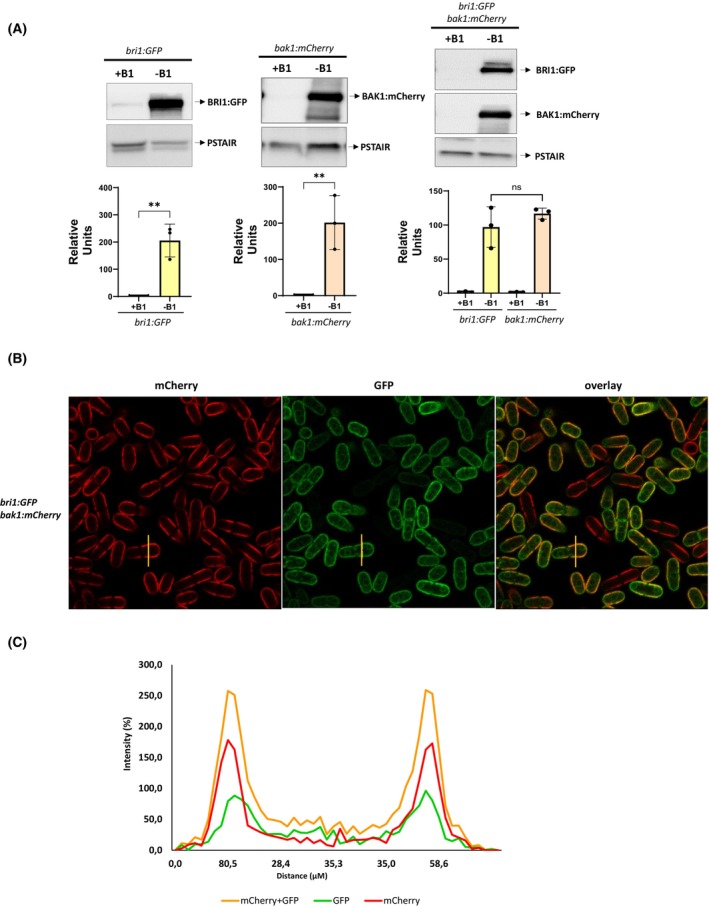
Expression and cellular localization of *Arabidopsis* Brassinosteroid Insensitive 1 (BRI1) and Brassinosteroid‐insensitive receptor‐Associated Kinase 1 (BAK1) receptors. (A) Total extracts expressing single (left and central panels) or double (right panel) BRI1:GFP and BAK1:mCherry proteins through the activation of the inducible *nmt* promotor of the plasmids pAV0751‐*bri1:GFP* and pAV0356‐*bak1:mCherry* in the absence of thiamine (−B1) during 17 h or repressing the *nmt* promotor in the presence of thiamine (+B1), were resolved in SD/PAGE and analysed by western blotting. Blot membranes were cut prior to antibody incubation to detect BRI1:GFP and BAK1‐mCherry proteins by anti‐GFP and anti‐mCherry antibodies, respectively. Anti‐Cdc2 (PSTAIR) was used as a loading control (upper panel). Band intensities were quantified and normalized to the loading internal control (anti‐cdc2/PSTAIR) to account for variability in protein loading, values are expressed as mean ± SE. Experiments were performed independently three times. **, *P*‐value < 0.01; ns, not significant, as calculated by unpaired Student's *t*‐test (low panel). (B) Cellular localization of BRI1:GFP and BAK1:mCherry expressed together as in (A) were detected by confocal fluorescence microscopy. A total of 200 cells were analysed and figure shows a selection of representative cells. (C) Plot profile, the cells of interest (yellow line in B) were selected to analyse the fluorescence intensity distribution of each fluorescence marker. The abscissa axis (*x*) represents the tangential distance (μm) of the fluorescence intensity distribution in the labelled cell, and the ordinate axis (*y*) represents the percentage of fluorescence intensity in that cell. The analysis was performed on 10 individual cell and the plot profile shows a representative one.

To determine the effect of biostimulants priming on growth activation through the steroid hormone receptor, *Arabidopsis thaliana* receptors BRI1 fused to GFP protein (BRI1‐GFP) and BAK1 fused to mCherry protein (BAK1‐mCherry) were cloned and integrated into the fission yeast genome under the control of the strong *nmt1* inducible promoter (Fig. S1). The single and double expression of the receptors, BRI1‐GFP and Bak1‐mCherry, upon induction is shown in Fig. 1A. Thiamine removal resulted in the high expression of both receptors in fission yeast cells, and the level of expression was essentially the same when the two genes were concomitantly induced (Fig. 1A).

Next, the cellular localization of the expressed plant receptors was analysed by microscopy. Both BRI1‐GFP and BAK1‐mCherry receptors were mainly localized to the plasma membrane, with only a slight signal detected in the cytoplasm, as has also been described in plant cells (Fig. 1B,C). From the microscopy image, a representative cell (yellow line) was selected to analyse the fluorescence intensity distribution of each fluorescence marker using a plot profile. As shown in Fig. 1C, the signal from both receptors perfectly matched the margin of the cell.

### Activation of BRI1‐BAK1 receptors

To investigate whether the membrane‐expressed receptors, BRI1 and BAK1, were functional and could respond to brassinosteroids, the activation of the MAPK pathway after treatment with brassinolide was analysed.

MAPK signalling is regulated by intracellular signalling induced by the brassinosteroid response in plants [45, 46]. Brassinolide (BL) was used in our experimental setup, the most active brassinosteroid member of the polyhydroxylated steroid hormone group, which is ubiquitous in almost all plant species [47].

**Fig. 2:**
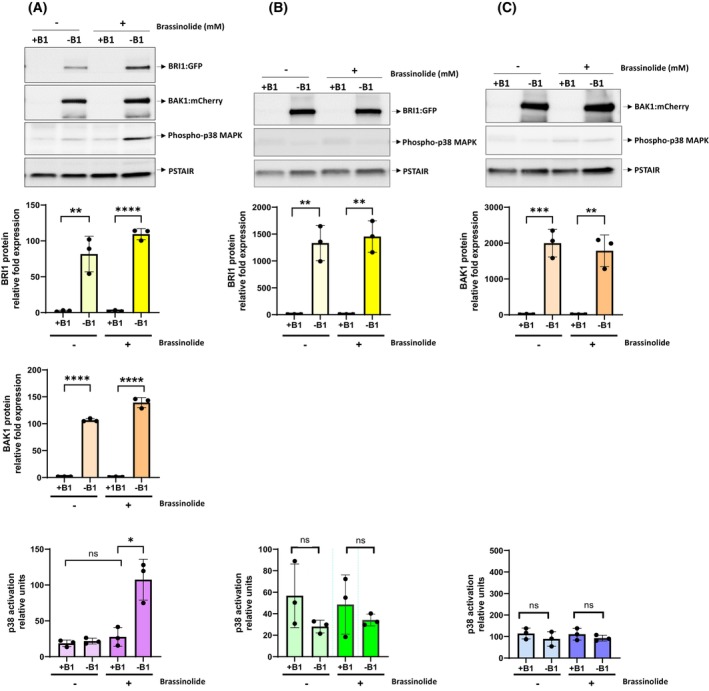
Activation of Brassinosteroid Insensitive 1 (BRI1)/Brassinosteroid‐insensitive receptor‐Associated Kinase 1 (BAK1) receptors. Total extracts expressing together (A) or single BRI1:GFP (B) and BAK1:mCherry (C) proteins through the activation of the inducible *nmt* promoter of the plasmids pAV0751‐*bri1:GFP* and pAV0356‐*bak1:mCherry* in the absence of thiamine (−B1) during 17 h or repressing the *nmt* promoter in the presence of thiamine (+B1), were resolved in SDS/PAGE and analysed by western blotting. Blot membranes were cut prior to antibody incubation to detect BRI1:GFP and BAK1‐mCherry proteins by anti‐GFP and anti‐mCherry antibodies, respectively, and the activated MAPK p38 by anti‐phospho‐p38 antibody. Anti‐Cdc2 (PSTAIR) was used as a loading control (upper panels). Quantification of relative protein levels and fold changes in expression of BRI1, BAK1 and Sty1 phosphorylation (detected by anti‐phospho‐p38 blot) was performed by analysis of western blots. Band intensities were normalized to the internal loading control (anti‐cdc2/PSTAIR) to account for variability in protein loading and values are expressed as mean ± SE. Statistical significance was determined using an unpaired Student's *t*‐test, as shown in the lower graph panels: *P‐value < 0.05; **P‐value < 0.01; ***P‐value < 0.001; ****P‐value < 0.0001; ns, not significant, as calculated by unpaired Student's *t*‐test (low graphs panels).

Treatment of cells overexpressing BAK1 and BRI1 with BL resulted in activation of the MAPK pathway, as shown by the enhanced phosphorylation of fission yeast MAPK Sty1 (Fig. 2A, fourth lane). Notably, neither the overexpression of the receptors in untreated cells nor BL treatment on its own resulted in a similar increase in MAPK signalling. We also considered whether the individual overexpression of either receptor could result in Sty1 MAPK activation upon BL treatment. As shown in Fig. 2B,C, overexpression of either BAK1 or BRI1 in the presence of BL did not activate Sty1. These results clearly show that the activation of Sty1 MAPK occurs only when both receptors are present and treated with BL.

### Gene expression profiles of BL‐activated receptors in fission yeast

To create a gene reporter assay that could reflect the activation of the brassinosteroid response, we first sought to determine which genes were differentially induced upon BL treatment in our model system. Hence, mRNA expression was analysed in cultures expressing the *Arabidopsis* BRI1‐BAK1 receptors, mock‐treated or treated with BL (Table 1), using mRNA‐Seq. More than 93% of the reads were obtained as unique maps, and mRNA expression was quantified and normalized for each sample.

**Table 1:** List of samples and treatments handled in the RNAseq analysis. Samples were distributed in six experimental groups, defined by variables as follows: WT (wild‐type strain not expressing receptors, untreated), WTBL [wild‐type strain not expressing receptors, treated with brassinolide (BL)], NBB [strain not expressing Brassinosteroid Insensitive 1 (BRI1) and Brassinosteroid‐insensitive receptor‐Associated Kinase 1 (BAK1) receptors, untreated], NBBBL (strain not expressing BRI1 and BAK1 receptors, treated with BL), IBB (strain expressing BRI1 and BAK1 receptors, untreated), IBBBL (strain expressing BRI1 and BAK1 receptors, treated with BL). Each experimental group was analysed by triplicate.

Sample ID	Receptor expression	Treatment
WT	Without receptors	No treatment
WTBL	Without receptors	BL treatment
NBB	No receptors expressed	No treatment
NBBBL	No receptors expressed	BL treatment
IBB	Expressed receptors	No treatment
IBBBL	Expressed receptors	BL treatment

**Fig. 3:**
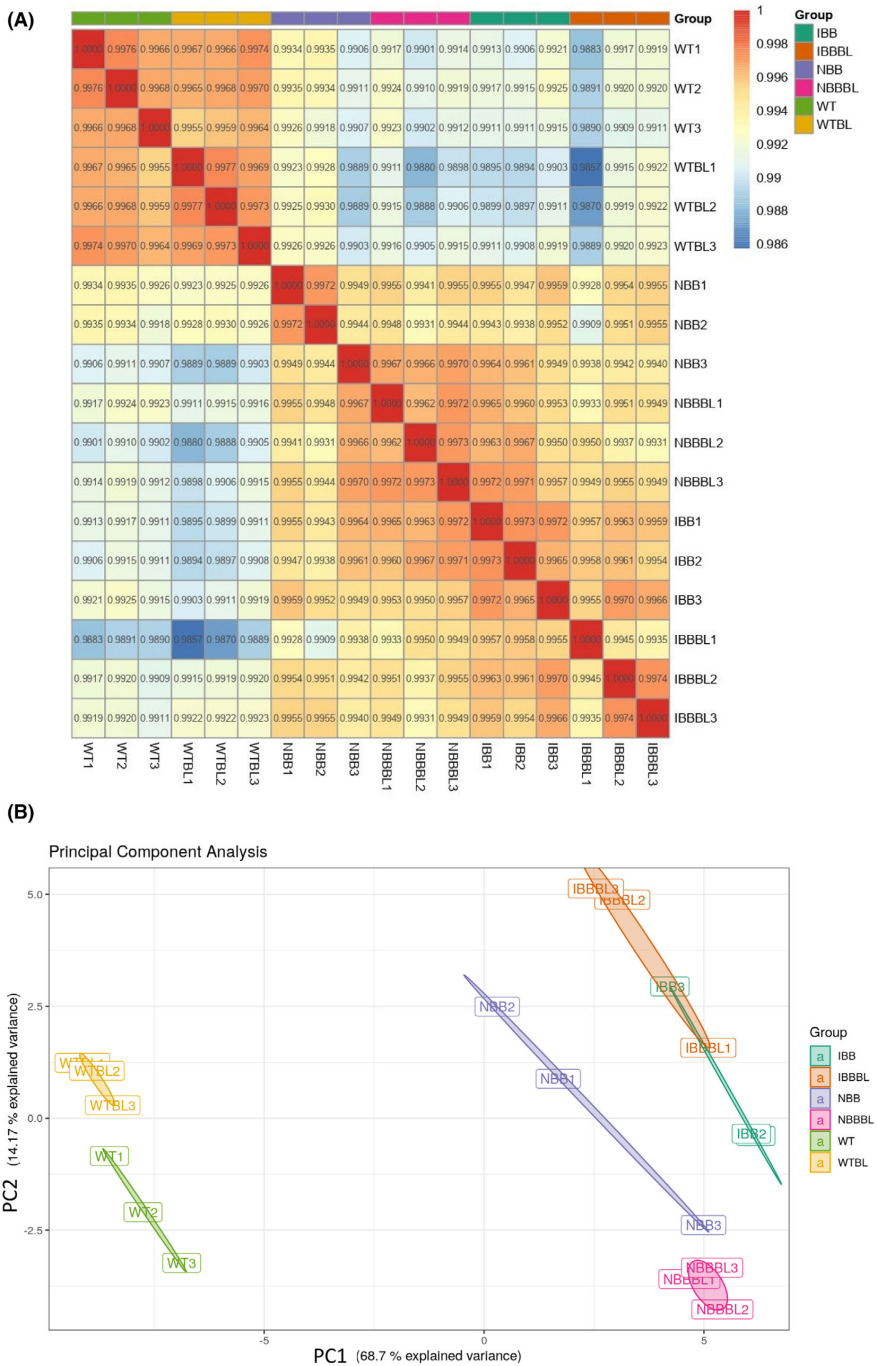
Gene expression correlations among all pairwise combinations of samples in the dataset. (A) Pearson correlation heatmaps and (B) Principal component analysis (PCA) of gene expression, among WT strain (*n* = 3), WTBL strain (*n* = 3), NBB strain (*n* = 3), NBBBL strain (*n* = 3), IBB strain (*n* = 3) and IBBBL strain (*n* = 3). Ratios of variance are shown as PCs. Strains WT: without receptors; no treatment. WTBL: without receptor; BL treatment. NBB: no receptors expressed; no treatment. NBBBL: no receptors expressed; BL treatment. IBB: expressed receptors; no treatment. IBBBL: expressed receptors; BL treatment.

Pearson correlation analysis of the mRNA‐Seq data showed that all samples were closely correlated within groups (Pearson correlation coefficient, *R* ^2^ = ~0.993; Fig. 3A). A single node transition was identified between the WT and IBB (induced BRI1‐BAK1 receptors) groups; and the *R* ^2^ tended to be larger between the WT group and the IBB than the IBBBL (induced BRI1‐BAK1 receptors treated with BL) group. The first and second principal components, PC1 and PC2, from 1000 genes explained 68.7% and 14.17% of the total variation in mRNA expression, respectively (Fig. 3B). The divergence of the first principal component (PC1) generally reflected the differences between the groups, with an evident divergence between the IBBBL and WT groups. Similar to the Pearson correlation results, on the PC1 axis, the NBB (no induced BRI1‐BAK1 receptors) and NBBBL (no induced BRI1‐BAK1 receptors treated with BL) groups were located between the WT and IBB/IBBBL groups.

**Fig. 4:**
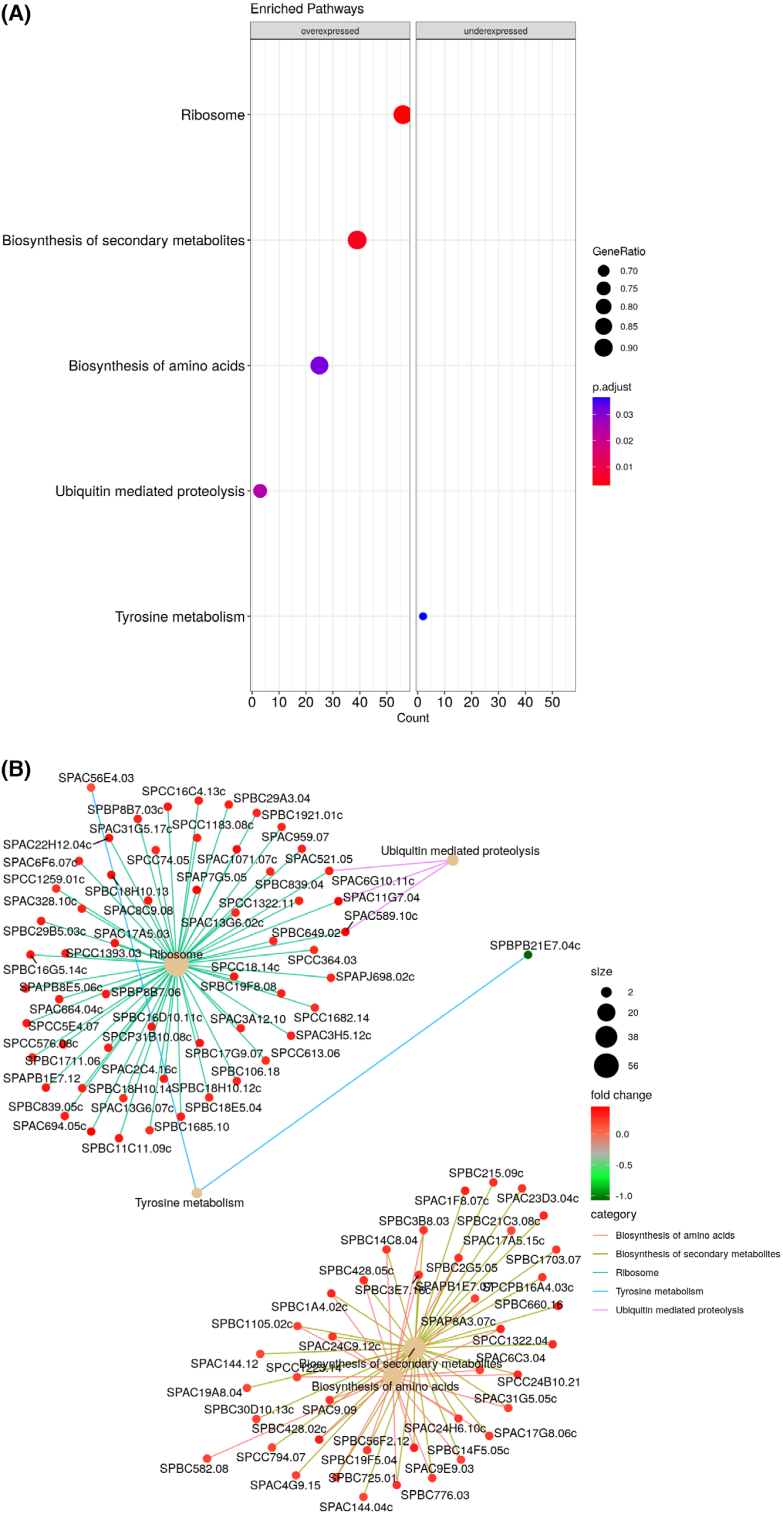
Gene Ontology (GO) and KEGG Pathway analysis. Differentially expressed genes reported by deseq2 (*P*‐value < 0.05) were used for Gene Set Enrichment Analysis among IBBBL (*n* = 3) and IBB (*n* = 3) cells. (A) Enrichment GO dot plot, shows the five most significantly pathways corresponding to upregulated (over‐expressed) or downregulated (under‐expressed) sets of genes. The colour represents the *P*‐values relative to the other displayed pathways (brighter red is more significant) and the size of the pathways represents the number of genes that are significant from the list. Gene ratio refers to the total number of genes in that pathway. (B) Category netplot between the genes associated with the top five most significant GO pathways and the fold changes of the significant genes associated with these pathways (colour). The size of the GO terms reflects the *P*‐values of the pathway, with the more significant terms being larger.

GO (Gene Ontology) and Kyoto Encyclopaedia of Genes and Genomes (KEGG) were used to identify the biological processes and signalling molecular pathways associated with differentially expressed genes (DEGs). Of the 4862 expressed genes detected, 274 were differentially expressed in the IBBBL group compared with IBB. Figure 4A shows the most significant pathways associated with differentially expressed genes. Four pathways were at the top of the entire ranked list of upregulated genes, and one cellular pathway was at the bottom of the ranked list corresponding to downregulated genes. The category netplot (Fig. 4B) depicts the linkages of genes and biological concepts as a network to visualize which genes are included in each pathway and which are shared between the pathways. The five most significantly enriched pathways were identified corresponding to over‐ and under‐expressed sets of genes. The four upregulated pathways were ribosome (KEGG PW:spo03010, *P* = 0.003, 60 genes), biosynthesis of secondary metabolites (KEGG PW:spo01110, *P* = 0.005, 42 genes), ubiquitin‐mediated proteolysis (KEGG PW:spo04120, *P* = 0.024, 4 genes), biosynthesis of amino acids (KEGG PW:spo01230, *P* = 0.031, 28 genes) and the downregulated pathway: tyrosine metabolism (KEGG PW:spo00350, *P*‐value = 0.036, 3 genes) (Fig. 4B).

Among the 274 expressed genes detected in IBBBL, 17 genes whose expression was significantly enhanced comparing IBBBL cells to IBB were selected (Table 2).

**Table 2:** Differentially expressed genes in response to BL treatment. List of genes differentially expressed between cells expressing Brassinosteroid Insensitive 1 (BRI1)‐Brassinosteroid‐insensitive receptor‐Associated Kinase 1 (BAK1) receptors treated with brassinolide (BL) and untreated. Bold formatting highlights the two selected genes.

Systematic ID	Gene name	Product
SPAC1783.02c	*vps66*	1‐acylglycerol‐3‐phosphate O‐acyltransferase Vps66
SPBC1348.07	none	*S. pombe* specific DUF999 protein family 6
SPCC1795.08c	*vid21*	NuA4 histone acetyltransferase complex subunit Vid21
**SPAC1F8.03c**	***str3***	**Plasma membrane heme transmembrane transporter Str3**
**SPBC660.05**	***wwm3***	**WW domain containing protein of unknown function**
SPBC18E5.11c	*edc3*	Enhancer of mRNA decapping Edc3
SPBPB8B6.03	*Fah1*	Fatty acid amide hydrolase Fah1
SPAC22F3.04	*mug62*	Acetyl‐CoA biosynthesis protein
SPAC977.09c	*plb4*	Phospholipase Plb4
SPCC1450.15	None	Pig‐F/3‐ketosphinganine reductase fusion protein
SPBC8D2.14c	*sed5*	SNARE Sed5
SPAC607.02c	None	Conserved fungal protein
SPBC18E5.10	None	Mitochondrial iron–sulfur cluster protein
SPCC1620.11	*nup97*	Nucleoporin Nic96 homologue
SPAC4G8.10	*gos1*	SNARE Gos1
SPBP8B7.28c	*stc1*	CLRC complex‐Argonaute linker protein Stc1
SPBC1271.06c	*mug96*	*S. pombe* specific protein

**Fig. 5:**
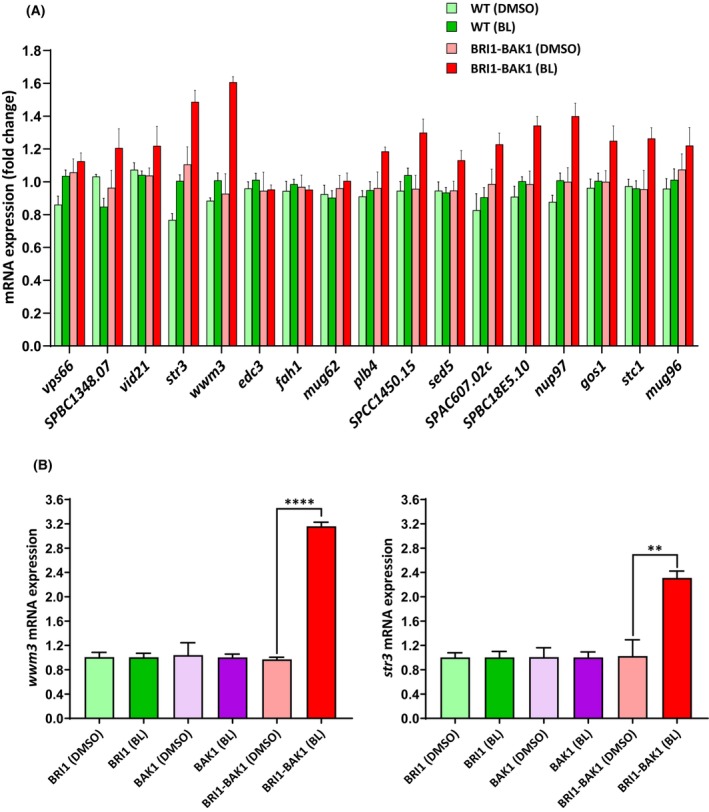
Gene expression of selected genes activated by brassinolide (BL) through Brassinosteroid Insensitive 1 (BRI1) and Brassinosteroid‐insensitive receptor‐Associated Kinase 1 (BAK1) receptors evaluated by RT‐PCR. (A) The relative mRNA of 17 differentially expressed genes identified in mRNA‐Seq analysis in *S. pombe* strains expressing and non‐expressing the BRI1‐BAK1 receptors, treated and non‐treated with BL, analysed by RT‐PCR method. (B) *str3* and *wwm3* mRNA expression in cells expressing both or individually BRI1 and BAK1 receptors, treated with BL. All gene expression values were normalized using the Ct value corresponding to the expression of *cdc2* as a housekeeping gene in the different experimental groups. Experiments were performed independently three times in triplicate. Gene expression values are expressed as mean ± SE. Statistical significance was determined using one‐way ANOVA multiple comparisons, **P‐value < 0.01; ****P‐value < 0.0001.

In order to corroborate the mRNA‐Seq results, the expression of the 17 selected genes was analysed by quantitative PCR assay (qPCR) under the same conditions. As shown in Fig. 5A, most of the selected genes exhibited differential expression. Of the 17 analysed genes, *str3* and *wwm3* showed the highest level of induction and were selected for further analysis. In contrast, no significant differences in expression were observed between wild‐type cells mock‐treated or treated with BL and the level of expression in untreated BRI1‐BAK1 overexpressing cells (Fig. 5A).

To demonstrate that the enhanced expression of *str3* and *wwm3* genes is due to BL‐induced activation through both BRI1 and BAK1 receptors simultaneously, the qPCR assay, including the strains expressing individually BRI1 and BAK receptors and treated with BL, was repeated. Higher expression of *str3* and *wwm3* was observed only in the strain expressing both BRI1 and BAK1 receptors treated with BL, but not in the strains expressing either one of the two receptors (Fig. 5B). This result demonstrated that the induction of *str3* and *wwm3* expression when cells were treated with BL was specifically dependent on the presence of both BRI1 and BAK1 receptors.

### BL‐dependent activation of *str3* and *wwm3* reporters

**Fig. 6:**
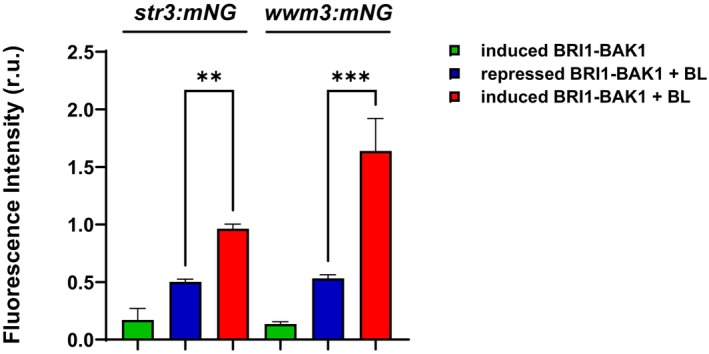
Activation of *str3:mNG* and *wwm3:mNG* reporters by BL. Cells expressing (induced) or not (repressed) Brassinosteroid Insensitive 1 (BRI1)‐Brassinosteroid‐insensitive receptor‐Associated Kinase 1 (BAK1) carrying *str3‐mNG* (left) or *wwm3‐mNG* (right) reporters were treated with BL during 3 h. The different cells were subjected to flow cytometry to measure the mNG levels related to the functional transcriptional activity of *str3* and *wwm3* promoters. Experiments were performed three times independently. Results are shown as the mean ± SE. Statistical significance was determined using two‐way ANOVA multiple comparisons; **P‐value < 0.01, ***P‐value < 0.001.

Having established that *str3* and *wwm3* transcription respond to BL exposure in cells overexpressing BRI1‐BAK1 receptors, the promoters of these genes were used to develop a reporter system to study the brassinosteroid response. To this end, the promoter regions of *str3* and *wwm3* were fused to the *mNeon Green* (*mNG*) coding sequence, and the two resulting constructs were independently integrated into the genome of the strain carrying BRI1 and BAK1 receptors tagged with 3HA and 13Myc epitopes, respectively (Fig. S2A,B). Thus, the transcriptional activity of the integrated reporters was linked to the recombinant expression of mNG, allowing for its detection by flow cytometry. In good agreement with the mRNA expression data, treatment with BL had a significant and specific effect on the activation of either *str3‐mNG* or *wwm3‐mNG* reporters in cells expressing the BRI1‐BAK1 receptors when compared to cells without the expression of the receptors (Fig. 6 and Fig. S3A,B). In addition, fluorescence values detected by flow cytometry for the *wwm3* reporter were higher than those for the *str3* reporter, confirming the qPCR results (Figs 5 and 6).

### Evaluation of biostimulants capacity to activate specifically BRI1‐BAK1 receptors

To test the developed system, we used three biostimulants from the manufacturer Fertinagro Biotech, referred to as E600, HBPAL and AA22, all derived from organic matter recycling. The composition of these biostimulants is detailed in Table 3. Among the three biostimulants, AA22 stands out due to its high concentration of free amino acids. In contrast, HBPAL and E600 contain similarly low levels of free amino acids. However, E600 presents approximately twice the nitrogen content of HBPAL, primarily attributable to the presence of peptides formed through protein hydrolysis.

**Table 3:** Biostimulants used in the work.

Biostimulants	Composition
E6‐0‐0 (E600)	Pig skin and hair processed by subcritical water and concentrated by evaporation. Nitrogen content is 6% and 3% of free amino acids
HBPAL	Pig blood processed by alkaline hydrolysis. Buffered and filtered. Composition, 3% of Nitrogen and 3% of free amino acids
Amino22 (AA22)	Pig blood hydrolysed with acid, buffered and filtered. It contains 5% total Nitrogen and 22% of free amino acids

To determine effective working concentrations for the biostimulation assays, we initially conducted dose–response experiments using a range of concentrations for each compound.

**Fig. 7:**
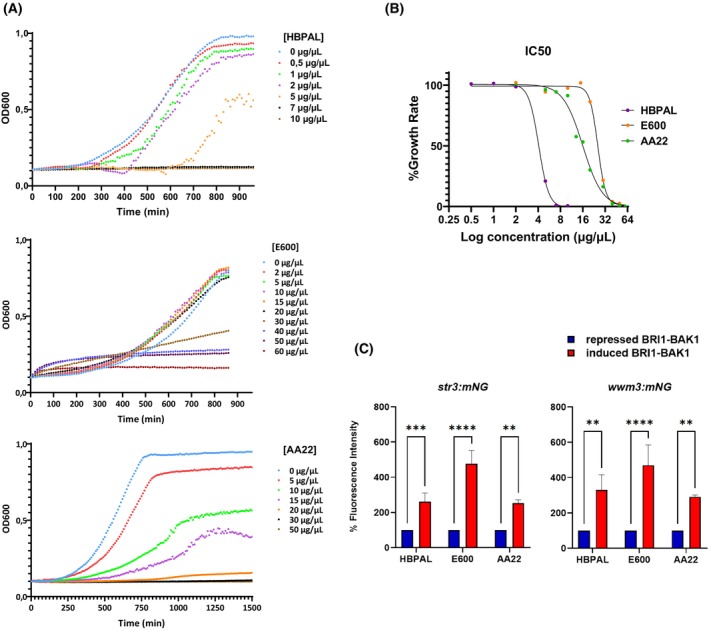
Capacity of HBPAL, E600 and AA22 to activate Brassinosteroid Insensitive 1 (BRI1)‐Brassinosteroid‐insensitive receptor‐Associated Kinase 1 (BAK1) receptors. (A) Yeast cells carrying repressed BRI1‐BAK1 receptors were grown to log phase and transferred to 96‐well plates. Indicated concentrations of HBPAL, E600 and AA22 biostimulants were added and optical density (OD) was monitored every 10 min for 16–20 h. (B) Dose dependence curves of yeast cells carrying repressed BRI1‐BAK1 receptors treated with the indicated biostimulants determined from OD at 20 h. (C) Cells not expressing (repressed) or expressing (induced) BRI1‐BAK1 carrying *str3‐mNG* (left) or *wwm3‐mNG* (right) reporters were treated with 5 μg·mL^−1^ E600, 2 μg·mL^−1^ HBPAL and 5 μg·mL^−1^ AA22 during 3 h. The different cells were subjected to flow cytometry to measure the mNG levels related to the functional transcriptional activity of *str3* and *wwm3* promoters. Experiments were performed three times independently. Results are shown as the mean ± SE. Statistical significance was determined using two‐way ANOVA multiple comparisons; **P‐value < 0.01, ***P‐value < 0.001, ****P‐value <  0.0001.

The IC50 of each biostimulant was defined by growing wild‐type *S. pombe* cells in a 96‐well plate with different concentrations of each biostimulant; cell growth was assayed by measuring the optical density (OD) every 10 min (Fig. 7A). After optimizing the growth curves, the highest concentration was observed at 7 μg·μL^−1^ for HBPAL, 60 μg·μL^−1^ for E600 and 30 μg·μL^−1^ for AA22 (Fig. 7A).

To determine the IC50 of each biostimulant, a dose‐dependent curve was generated by plotting the growth rate in % (the slope of the exponential growth phase) normalized to that of untreated cells as a function of the log concentration of the biostimulant (Fig. 7B). Table 4 shows the IC50 values of the three biostimulants: HBPAL 4 μg·μL^−1^, E600 25 μg·μL^−1^ and AA22 16 μg·μL^−1^.

**Table 4:** IC50 (μg·μL^−1^) of the biostimulants.

	HBPAL	E600	AA22
Best‐fit values			
Bottom	−0.7202	1.229	−0.6554
Top	100.7	99.19	100.7
**IC50**	**4.073**	**25.43**	**15.99**
HillSlope	−6.424	−8.118	−3.274
logIC50	0.6099	1.405	1.204
Span	101.5	97.96	101.3
Goodness of fit			
Degrees of freedom	3	6	8
***R* squared**	**0.9997**	**0.9974**	**0.9892**
Sum of squares	4.629	52.40	210.7
Sy.x	1.242	2.955	5.132
Constraints			
IC50	IC50 > 0	IC50 > 0	IC50 > 0
Number of points			
# of *X* values	7	16	16
# *Y* values analysed	7	10	12

The selected genes were highlighted in bold.

To assess the ability of selected biostimulants to activate the BRI1–BAK1 receptor complex, we treated different clones expressing BRI1–BAK1 receptors and carrying either the *str3*‐mNG or *wwm3*‐mNG reporters with HBPAL (2 μg·μL^−1^), E600 (5 μg·μL^−1^) or AA22 (5 μg·μL^−1^). These concentrations were selected based on preliminary dose–response experiments that identified the lowest effective concentrations capable of eliciting a robust and reproducible biological response without inducing toxicity. Although IC50 values were previously estimated (HBPAL: 4 μg·μL^−1^; E600: 25 μg·μL^−1^; AA22: 16 μg·μL^−1^), they were not used directly to guide treatment doses. Instead, the concentrations applied in the biostimulation assays reflect empirically optimized conditions tailored to maximize receptor activation while maintaining cell viability. The transcriptional activation of the *str3* and *wwm3* reporters was determined after 3 h of biostimulant exposure by flow cytometry. The activation effect of the biostimulants was referred to as the negative control, consisting of cells treated with the same concentration of the indicated biostimulant; however, the expression of BRI1‐BAK1 receptors was repressed. As shown in Fig. 7C (and Fig. S4A,B), E600 was the biostimulant with the highest capacity to activate BRI1‐BAK1 receptors and therefore has a greater capacity to induce plant growth and development. HBPAL and AA22 also had a significant effect on the activation of the BRI1‐BAK1 receptors, albeit to a lesser extent. Both treatments showed a biostimulant capacity approximately half that of E600 (Fig. 7C and Fig. S4A,B).

### Effect of biostimulants on *S. pomb*e cell growth

**Fig. 8:**
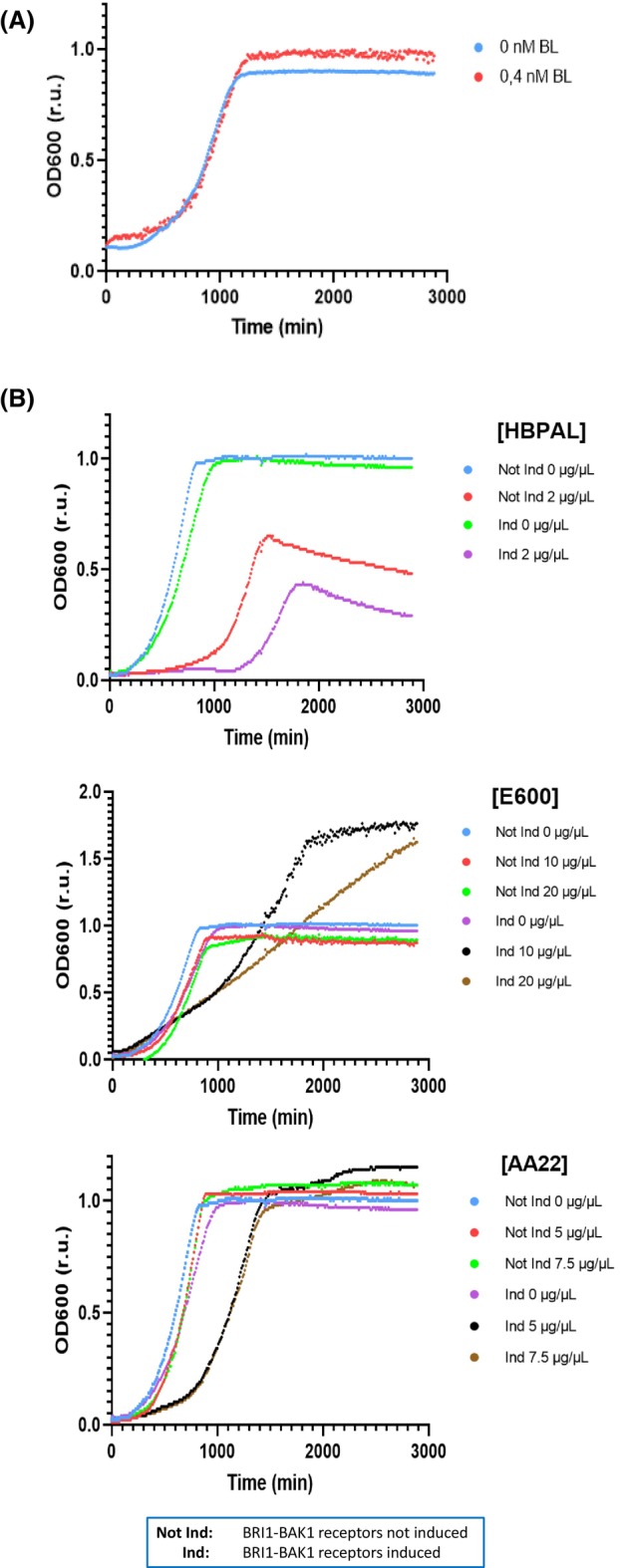
Effect of HBPAL, E600 and AA22 biostimulants on *S. pombe* cell growth. (A) Growth curves of cells expressing Brassinosteroid Insensitive 1 (BRI1)‐Brassinosteroid‐insensitive receptor‐Associated Kinase 1 (BAK1) receptors in the presence of BL (0.4 nm BL) or absence (0 nm BL) referred to the same cells with BRI1‐BAK1 expression repressed. (B) Growth curves of cells expressing (induced) or not (not induced) BRI1‐BAK1 receptors in the presence of indicated concentrations of HBPAL, E600 and AA22 biostimulants. In all analyses, cells were grown to log phase and transferred to 96‐well plates. Indicated concentrations of BL or biostimulants were added, and optical density was monitored every 10 min for 48 h. Each point shows the average of four replicates.

To confirm the above results, in which the E600 biostimulant had the highest effect on BRI1‐BAK1 gene reporter activation among the biostimulants tested, the growth effect of the biostimulants on *S. pombe* cells was analysed by examining cell proliferation by measuring the OD every 10 min (Fig. 8). First, the analysis of BL on *S. pombe* cell growth revealed a moderate but consistent increase in cell growth (Fig. 8A). The same analysis was performed with biostimulants HBPAL, E600 and AA22, and as Fig. 8B shows, E600 induced a high level of proliferation in *S. pombe* cells at both concentrations tested (Fig. 8B, middle panel). In contrast, neither HBPAL nor AA22 biostimulants showed an increase in proliferation compared with control cells (cells treated with repressed receptors, or untreated cells with activated receptors) (Fig. 8B, upper and lower panel). In addition, the HBPAL biostimulant displayed a toxic effect on *S. pombe* cell proliferation over time (Fig. 8B, upper panel).

### Growth chamber trials of the biostimulants

**Fig. 9:**
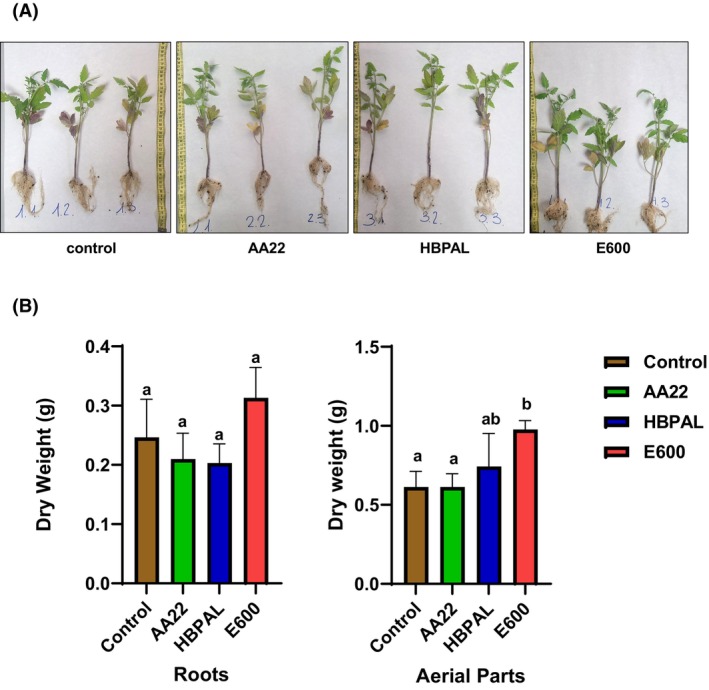
Effect of the biostimulants HBPAL, E600 and AA22 on the growth of *Solanum lycopersicum* L. (A) Six‐day‐old seedlings without treatment (control) and treated with AA22, HBPAL and E600. (B) Dry weight of root and shoot tissues following treatment with the indicated biostimulants. Bars with the same letter (a o b) are not significantly different according to Tukey's Honest Significant Difference (HSD) test (*P*‐value ≤ 0.05). Gene expression values are expressed as mean ± SE.

Controlled growth chamber experiments were conducted using *Solanum lycopersicum* L. (tomato) to evaluate the impact of biostimulant treatment on key plant growth parameters. A conventional granular 15‐15‐15 fertilizer was applied to all plants 1 week before biostimulant application. Plants were harvested 6 days after treatment (Fig. 9A), and samples were oven‐dried at 70 °C until constant weight to assess dry biomass. Results are shown in Fig. 9B. Significant differences were found for the aerial parts, corroborated by an analysis of variance, ANOVA (Fig. 9B). Tukey's *post hoc* analysis determined that the E600 treatment exhibited significant differences compared with the control, with a 95% confidence interval, indicating that it has a strong biostimulant capacity for *in vivo* growth under growth chamber conditions and correlates with results with the developed method and yeast growth analysis.

## Discussion

The present study was motivated by the need to deepen our understanding of biostimulant functions to optimize their efficacy. Despite considerable efforts, elucidating the mechanisms of action of these products remains particularly challenging [48]. This complexity arises primarily from the heterogeneous nature of biostimulants, which are derived from a wide array of biological and inorganic sources [4], including protein hydrolysates of agro‐industrial by‐products from both plant and animal origins. As a result of their diverse compositions, it is unlikely that biostimulants share a common mode of action. Accordingly, there is a critical demand for robust and sensitive tools capable of identifying new biostimulants, characterizing their biological activities, and elucidating their mechanisms of action.

In this context, we established a novel system designed to classify biostimulants based on their capacity to activate specific plant receptors associated with defined biological functions. Specifically, we focused on the activation of the BRI1‐BAK1 receptor complex, a key regulator of plant growth and development.

The *Arabidopsis* BRI1 and BAK1 receptors were heterologously expressed in *S. pombe* (fission yeast). Upon induction, both receptors localized to the plasma membrane and could be activated by exogenous application of brassinolide (BL). BL treatment resulted in the activation of a specific transcriptional programme dependent on the presence of BRI1‐BAK1 receptors. Functional enrichment analysis of mRNA‐Seq data revealed that the upregulated genes were predominantly involved in ribosomal biogenesis and amino acid metabolism, pathways typically associated with enhanced protein synthesis during growth.

**Fig. 10:**
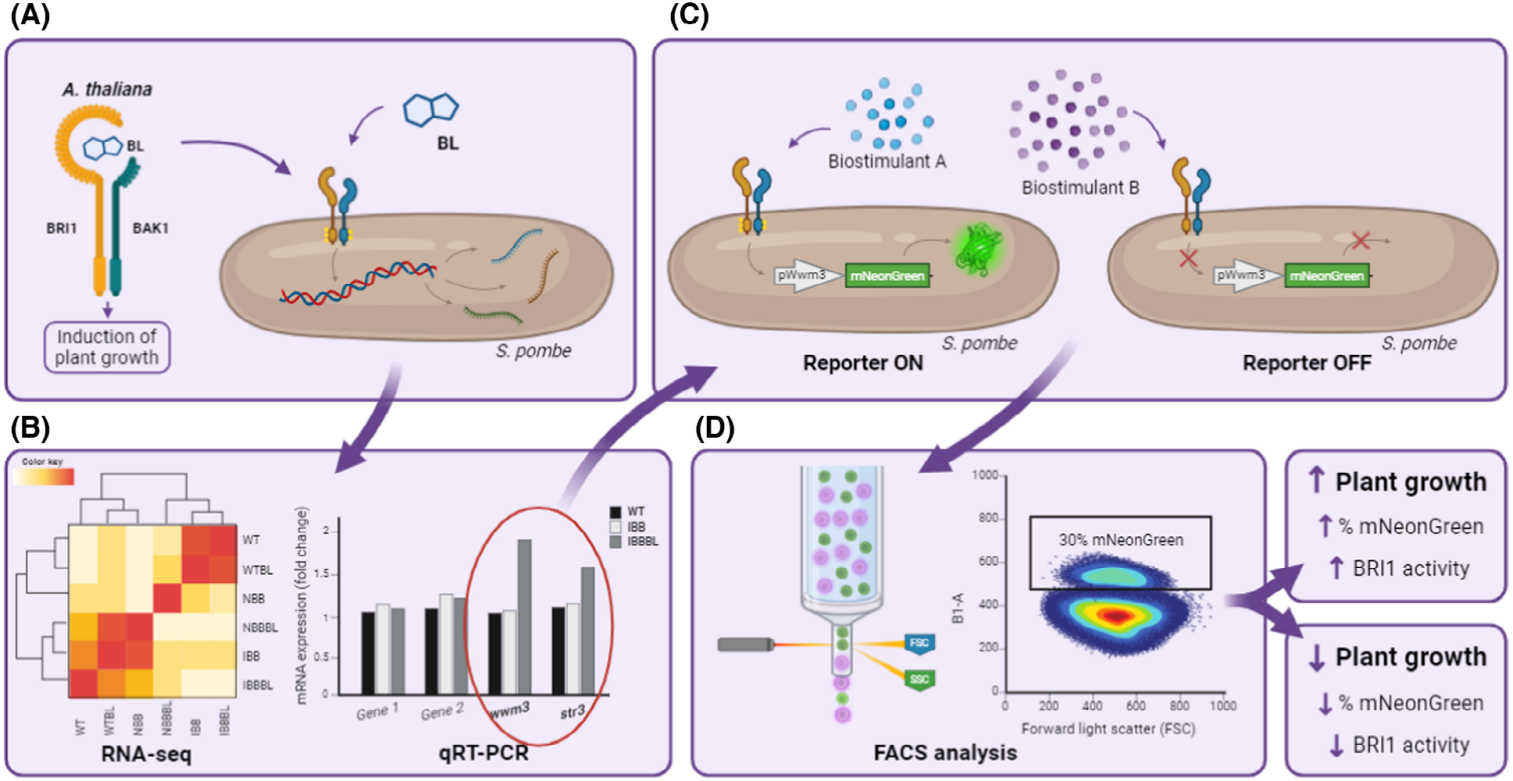
Summary of the method developed in this study. (A) Cloning of *A. thaliana* Brassinosteroid Insensitive 1 (BRI1)‐Brassinosteroid‐insensitive receptor‐Associated Kinase 1 (BAK1) receptors in fission yeast. (B) RNAseq analysis of BIK1‐BAK1 activation by BL and selection of upregulated expressed genes. (C) Generation of reporter gene‐GFP activated through BRI1‐BAK1 activation and evaluation of different biostimulants. (D) Detection of reporter activation by FACS analysis. Classification of biostimulants by their qualitative and quantitative capacity to activate BIK1‐BAK1 receptors. This figure was partially created with BioRender.com.

Among the 17 genes specifically induced by BL treatment in BRI1‐BAK1‐expressing cells, two genes, *str3* and *wwm3*, were selected as biomarkers due to their strict dependence on receptor activation. Neither gene was induced in wild‐type cells exposed to BL, nor in untreated receptor‐expressing cells, thereby establishing their specificity and utility as molecular indicators of BRI1‐BAK1 activation (Fig. 10).

This newly developed method proved effective in assessing the biostimulatory potential of different compounds. Among the biostimulants tested, E600, which is enriched in structured peptides, exhibited the highest receptor activation capacity. AA22 and HBPAL showed approximately 50% of the activation observed with E600. Notably, AA22, composed primarily of free amino acids, nonetheless elicited a measurable activation response, highlighting the system's high sensitivity in detecting even modest receptor activation events.

To validate these findings in plants, growth chamber experiments were conducted using *Solanum lycopersicum* L (tomato). Consistent with the yeast‐based assays, E600 treatment resulted in significantly greater biomass accumulation compared with HBPAL and AA22 treatments. These results align with the receptor activation data and support the biological relevance of the yeast‐based assay.

Collectively, our findings demonstrate that fission yeast engineered to express plant receptors constitutes a powerful platform for the functional characterization and screening of biostimulants. This system offers specificity, sensitivity and quantitative resolution, thereby providing a valuable tool for both research and industrial biostimulant development.

## Conclusion

In this study, we developed a novel fission yeast‐based system for the functional characterization of biostimulants through the activation of the *A. thaliana* BRI1‐BAK1 receptor complex. The system demonstrated high specificity and sensitivity, enabling the detection and quantification of receptor activation induced by different biostimulant formulations. Among the tested products, E600, characterized by a high peptide content, exhibited the strongest activation capacity, consistent with enhanced growth responses observed in *Solanum lycopersicum*.

The ability of the system to detect activation even in biostimulants composed predominantly of free amino acids (e.g. AA22) highlights its robustness and broad applicability. Moreover, the identification of specific molecular markers (*str3* and *wwm3*) linked to receptor activation provides a reliable readout for high‐throughput screening efforts.

Furthermore, our method offers the potential to facilitate the characterization of the diverse functions of biostimulants by enabling the activation and study of other plant receptors beyond BRI1‐BAK1.

Overall, our findings support the use of *S. pombe* as a versatile and effective platform for the prescreening and mechanistic study of biostimulants, ultimately contributing to the development of more targeted and effective agricultural products.

## Materials and methods

### Plasmid construction

All plasmids were generated using standard restriction enzyme cloning (Restriction Enzymes; Thermo Fischer Scientific, Waltham, MA, USA). The list of all plasmids used in the study is available in Table S1. The backbone plasmids pAV0751, pAV0607 and pAV0356 were created by Dr Martin [49] and were acquired from the ‘National BioResource Project Yeast Genetic Resource Center’ (NBRP/YGRC. http://yeast.lab.nig.ac.jp/).

The *Arabidopsis* BRI1 open reading frame (ORF) without stop codon was removed from pUCIDT‐*bri1* plasmid (produced by ‘Integrated DNA Technologies’) using *SalI/PacI* restriction enzymes and cloned into the pAV0751 plasmid, containing uracil (*Ura4* ^AfeI^) as a marker for integration in yeast and a C‐terminal GFP tag, to obtain pAV0751‐*bri1:GFP* plasmid (Fig. S1A).

The *Arabidopsis* BAK1 ORFs without stop codon was amplified by PCR from pUCIDT‐*bak1* plasmid (produced by ‘Integrated DNA Technologies’) using *bak1‐Xho1*‐Fwr and *bak1‐EcoRI*‐Rev primers (for primer sequences, see Table S2). The resultant PCR product was inserted into the pAV0751.1 plasmid, containing a C‐terminal mCherry tag, to obtain pAV0751.1‐*bak1‐mCherry* plasmid. pAV0751.1 plasmid was obtained by sub‐cloning *mCherry‐RitC‐terminator* ^*ScSDH1*^ DNA fragment into the pAV0751 plasmid, using *SacI/EcoRI* restriction enzymes. *mCherry‐RitC‐terminator* ^ScADH1^ DNA fragment was extracted from the pAV0607 plasmid by using the same enzymes. The p^nmt1^‐*bak1‐mCherry*‐*RitC‐terminator* ^ScADH1^ DNA fragment was removed from pAV0751.1‐*bak1:mCherry* plasmid using *ApaI/SacI* restriction enzymes and cloned into the pAV0356 plasmid, containing Adenine (*Ade6* ^PmeI^) as a marker for integration in yeast, to obtain pAV0356‐*bak1:mCherry* plasmid (Fig. S1A).

pAV0751‐*bri1‐3HA* and pAV0356‐*bak1:13Myc* plasmids were obtained by replacing the GFP and mCherry tags with the 3HA and 13Myc tags from the pAV0751‐*bri1:GFP* and pAV0356‐*bak1:mCherry* plasmids, using *SalI/NotI* and *NruI/NotI* restriction enzymes, respectively (Fig. S2A).

The sequences immediately upstream of the START codons of the *str3* and *wwm3* genes were used to design reporter plasmids carrying the *mNeon green (mNG)* gene and *leu1* gene as a marker. The *str3* promoter includes, in addition to the 608 bp of the promoter region, 41 bp of the 5′ UTR (p*str3*); while the *wwm3* promoter contains 500 bp of the promoter region plus 279 bp of the 5′ UTR (p*wwm3*). The p*str3* and p*wwm3* promoters were cloned using *PstI/NcoI* restriction enzymes to obtain p*str3‐mNG* and p*wwm3‐mNG* plasmids (Fig. S2B).

### Yeast strains

The *S. pombe* strains used in this work are listed in Table S3.

Fission yeast transformations were performed by either standard lithium acetate transformation or electroporation protocols [50]. One microgram of the linearized plasmid was used per transformation. Unless otherwise indicated, plasmids were linearized with a single restriction enzyme present in between the two homology regions (e.g., *AfeI* for pURA4^AfeI^ and *PmeI* for pAde^PmeI^).

The backbone *S. pombe* strain JM1058, carrying the *his7‐366 leu1‐32 ade6‐m210 ura4‐d18* mutations, was used to transform linearized plasmid and select for prototrophs.

Multiple transformants were analysed by western blot and fluorescence microscopy to verify correct plasmid integration and expression.

### Growth conditions and reagents

The *S. pombe* strains were routinely grown by shaking at 30 °C in rich yeast extract plus supplement medium (YES) or Edinburgh minimal medium (EMM2) with 3% glucose and supplemented with adenine, leucine, histidine, uracil and lysine (225 mg·L^−1^) [51].

For western blotting and fluorescence microscopy, cells were precultured overnight in liquid EMM2 and then again subcultured overnight to reach exponential phase. Repression of the *nmt* promoter was achieved by supplementing thiamine (B1) at a final concentration of 5 μg·mL^−1^. Activation of the *nmt* promoter was achieved by growing cells in a free medium for a minimum of 17 h.

Cells were treated with 0.4 mm Brassinolide (BL) (CAS 72962‐43‐7; Santa Cruz Biotechnology) and grown for 90 min.

The biostimulants, produced by Fertinagro Biotech and referred to as E600, HBPAL and AA22, were derived from organic matter recycling. Their compositions are detailed in Table 3. As part of the manufacturer's protocol, the contents of free amino acids, as well as total and organic nitrogen, were analysed to guide the formulation of the biostimulant fertilizers. These analyses were conducted in the company's quality control laboratory, following standardized operating procedures (Table 3).

### Differentially expressed genes analysis by mRNA‐sequencing

Total RNA from *S. pombe* strains expressing the BRI1 and BAK1 receptors and treated with 0.4 mm BL for 90 min was extracted with the miRNeasy Mini Kit (Qiagen, Hilden, Germany), following the manufacturer's protocol. A NanoDropTM 1000 Spectrophotometer (Thermo Scientific) was used to determine the final RNA quantity. Three independent biological replicates were performed for mRNA‐Seq analysis. Differential expression analysis among the samples using Illumina NovaSeq 6000 technology (Illumina, Inc., CA, USA) and bioinformatic analysis was performed by Microomics Systems S.L. (https://www.microomics.com/).

### Quantitative real‐time PCR

To validate the mRNA‐Seq results, samples from *S. pombe* strains expressing the BRI1 and BAK1 receptors and treated with 0.4 mm BL for 90 min were subjected to the qRT‐PCR. The total RNA of the collected samples was extracted following the above‐mentioned method. cDNA was synthesized from 1.0 μg of total RNA primed with a random hexamer using a high‐capacity cDNA kit (Applied Biosystems, USA). RNA concentration, purity and integrity (RIN) are shown in Table S4. SYBR Green‐based quantitative PCR was performed using the ViiA7 Real‐Time PCR System (Applied Biosystems Corp., USA). All primers listed in Table S2 were obtained from Integrated DNA Technologies (Leuven, Belgium). Three independent biological replicates were generated, and three measurements were performed on each replicate. The level of expression was calculated according to the relative quantification method, considering the average cycle threshold (Ct) value of all control samples as a reference. All PCR reactions were normalized using the Ct value corresponding to the expression of *cdc2 as a* housekeeping gene. Relative quantification was performed using quantstudio™ Software.

### Western blot analysis

Fission yeast samples were homogenized in lysis buffer [67 mm Tris–HCl (pH 6.8); 2% SDS] and disrupted using the Mini‐Beadbeater‐16 (BioSpec Products, Inc., OK, USA). The quantity of whole protein was determined by using a DC™ Protein Assay Kit (Cat. no.: 5000111; Bio‐Rad). Thirty micrograms of whole protein extract was electrophoresed under reducing conditions by SDS/PAGE on Bolt™ 4–12% precast Bis‐Tris Plus Mini protein gel (Cat. no.: NW04120 Box; Invitrogen) and transferred to a nitrocellulose membrane by use of iBlot™ 2 Transfer Stacks (Cat. no.: IB23001; Invitrogen). After blocking with TBST plus 5% non‐fat milk or 5% BSA, membranes were incubated with primary antibodies, including anti‐Phospho‐p38 MAPK [Thr180/Tyr182] (diluted 1 : 1000, Cat. no.: 9211; Cell Signalling Technology), anti‐GFP (diluted 1 : 2000, Cat. no.: ab290; Abcam), anti‐mCherry (diluted 1 : 1000, Cat. no.: ab167453; Abcam), anti‐c‐Myc [9E10] (diluted 1 : 500, Cat. no.: 13‐2500; Invitrogen), anti‐HA [12CA5] (diluted 1 : 1000, Cat. no.: 11666606001; Roche) and anti‐Cdk1/Cdc2 (PSTAIR) (diluted 1 : 5000, Cat. no.: 06‐923; Sigma‐Aldrich) at 4 °C overnight. The membrane was incubated with horseradish peroxidase‐conjugated secondary antibodies at room temperature for 1 h. A WesternBright™ Quantum detection kit (Cat. no.: K‐12042‐D20; Advansta) was used to detect bands.

### Quantification of western blot experiments and reproducibility of results

Densitometric quantification of western blot was performed using imagej. The desired bands plus background were drawn as rectangles, and a profile plot was obtained for each band (peaks). To minimize the background noise in the bands, each peak floating above the baseline of the corresponding peak was manually closed off using the straight‐line tool. Finally, measurement of the closed peaks was performed with the wand tool. Relative units for BRI1 and BAK1 expression, and Sty1 activation were estimated by determining the signal ratio of the corresponding anti‐GFP (BRI1 expression), anti‐mCherry (BAK1 expression) and anti‐phospho‐p38 (Sty1 activation) blots with respect to the anti‐cdc2/PSTAIR (internal control). Unless otherwise stated, results shown correspond to experiments performed as biological triplicates. Mean relative units ± SE and/or representative results are shown. *P‐*values were analysed by unpaired Student's *t*‐test.

### Localization of receptors by confocal fluorescence microscopy

*S. pombe* cells expressing the Bri1:GFP‐Bak1:mCherry fusion proteins were grown in an appropriate culture medium in the presence and absence of thiamine. For live‐cell imaging of growing cells, *S. pombe* cells were collected by centrifugation at 300 ***g*** for 5 min, suspended in EMM2 medium (an optically clear and colourless medium) and immediately immobilized on the coverslip of a glass‐bottom dish.

For coating the surface of the coverslip for immobilization of the cells, a gridded glass‐bottom dish (a plastic culture dish with a gridded coverslip was used instead of a regular coverslip, attached to the opening in the bottom of the dish) (Cat. no.: 80826; Ibidi, Germany), and coated the surface of the coverslip with lectins to immobilize the yeast cells. Fifty microlitres of 1 mg·mL^−1^ aqueous concanavalin A (Wako Pure Chemical Industries, Japan) was used to coat the surface of the coverslip [52]. First, the lectin solution was spread over the entire surface of the coverslip. Then, after 1 min, most of the lectin solution was removed by pipetting, and the solution left on the glass surface was completely air‐dried overnight. After drying, 100 μL of cell suspension (approximately 1–5 × 10^6^ cells·mL^−1^) was placed on the coated coverslip as a droplet and incubated for 5 min at room temperature. The excess cells were removed; finally, they were incubated for a further 5 min to ensure fixation of the cells on the glass coverslip. Subsequently, 400 μL of EMM2 medium was added to avoid desiccation during observation.

GFP and mCherry fluorescence image series of 4 fields per seeded well were selected and recorded every 10 s for 1–2 h, using the Carl Zeiss LSM 880 Airyscan confocal microscope. Zeiss zen software was used to set up the microscope. Using a 63× immersion objective and with an image resolution of 512 × 512 pixels, images were acquired one by one. The resulting images were saved as stacks (one per point).

The imagej software (ImageJ.org.) was used to analyse microscopy images. Using the ‘Plot profile’ tool, the cells of interest were selected to analyse the fluorescence intensity distribution of each marker. With the obtained data, a coordinate plot was created.

### Liquid culture assay in 96‐well plate

To conduct the liquid culture assay, a logarithmic phase culture was adjusted to an optical density (OD_600_) of 0.1, as measured by a conventional spectrophotometer. Using a multichannel pipette, 100 μL of the adjusted culture was dispensed into each well of a sterile 96‐well assay plate. Biostimulants were added to the prepared 96‐well plate at different dilution series.

For continuous OD measurement of culture, the assay plate, covered with a lid, was positioned in a Synergy2 BioTek plate reader. The cells were maintained at 32 °C with gentle agitation. Absorbance readings at 600 nm were recorded every 10 min. The plates were removed after 16 h, and final OD measurements were taken.

### Half maximal inhibitory concentration (IC50) measurement

IC50 values were calculated from the variation of growth rates when the cells were treated with the biostimulants at different concentrations [53, 54]. The slopes of the exponential growth phase, occurring approximately between 500 and 700 min, were used to calculate growth rates. This was accomplished by applying a simple linear regression model to the collected data using graphpad prism. The percentage growth rate was then plotted against chemical concentration. To determine IC50 values, which represent the midpoint between maximum and minimum inhibition, a sigmoidal dose–response curve (with variable slope and four parameters) was employed. This curve was generated using graphpad prism and utilized the following sigmoidal equation:

$$y=\text{Bottom}+\frac{\mathrm{Top}-\text{Bottom}}{1+{\left(\frac{\mathrm{IC}50}{X}\right)}^{\text{HillSlope}}}$$

where top is the maximal growth rate for the culture at a given biostimulant concentration; bottom is the minimal growth rate.

### Spectral flow cytometry analysis

*S. pombe* cells expressing BRI1‐BAK1 receptors carrying the *str3‐mNG* and *wwm3‐mNG* integrated reporters were grown in an appropriate culture medium in the presence or absence of thiamine. After 17 h, cells were treated with the different biostimulants and grown for 3 h. Cells were collected by centrifugation at 300 ***g*** for 5 min and resuspended in PBS. After that, cells were centrifuged as before, carefully flicked off the supernatant, and resuspended in 1 mL of PBS for acquisition in Cytek Aurora flow cytometer (5‐laser; 355, 405, 488, 561 and 640 nm) on the Cytometry Unit from Scientific and Technological Centres (CCiTUB), using the spectroflo Software v2.2.0.2 and taking care to meet the following acquisition criteria: (a) Cytek assay settings are used as a starting point for instrument setup, (b) the scatter profiles of cells are on scale and the FSC area scaling factor (ASF) is optimized, (c) all tubes are recorded with the same fluorescence gain settings for each detector, (d) sufficient events are recorded to find a clear positive signal (100 000 total events for each positive and negative population). The flow cytometry results were analysed using flowjo™ v10.8 Software (BD Life Sciences).

### Plant material and growth conditions

Seeds of *Solanum lycopersicum* L. cv Roma were germinated in an inert coconut fibre substrate under controlled growth chamber conditions. The chamber was maintained at 26 °C with a photoperiod of 14 h light and 10 h dark. Irrigation was applied on demand.

A conventional granular fertilizer (15‐15‐15) was applied to all plants 1 week prior to the application of biostimulants at a rate equivalent to 5 kg·ha^−1^.

Six days after the application of the biostimulants, plants were harvested, and samples were processed to determine their dry weight. Samples were oven‐dried at a constant temperature of 70 °C for 48–72 h, or until reaching a constant weight.

### Statistical analysis

Data are presented as the mean and standard error of the mean (±SE) of at least three independent experiments (biological replicates). Statistical differences were determined by one‐way analysis of variance (ANOVA) for multiple datasets, followed by Tukey's Honest Significant Difference (HSD) test when comparisons were performed to a single control. Statistical significance was defined as *P* < 0.05. Data were analysed using the prism 8.0 software (GraphPad). Further statistical details can be found in the figure legends.

## Conflict of interest

The authors declare no conflict of interest.

## Author contributions

RA, TY and IS contributed to the conception and design of the study. RA, SA and IS acquired the funding. MM, MR‐F, RD‐P, PQ, EC, SA and SL‐A performed the research. MM, SL‐A and RA wrote the original draft of the manuscript. MM, MR‐F, RD‐P and RA prepared the figures. MM, MR‐F, RD‐P and TY prepared the graphs. All authors contributed to the article and approved the submitted version.

## Supporting information


**Fig. S1.** Plasmid maps of *BRI1:GFP* and *BAK1:mCherry* receptors integrated in *S. pombe* cells.
**Fig. S2.** Plasmid maps of *BRI1:3HA* and *BAK1:13Myc* gene receptors (A) and reporters *str3:mNG* and *wwm3:mNG* (B).
**Fig. S3.** Expression of *BRI1:3HA* and *BAK1:13Myc* gene receptors and activation of *str3:mNG* and *wwm3:mNG* reporters by BL.
**Fig. S4.** Activation of *str3:mNG* and *wwm3:mNG* reporters by HBPAL, E600 and AA22 biostimulants.
**Table S1.** List of plasmids used in the study.
**Table S2.** List of primers used in the study.
**Table S3.** List of *S. pombe* strains used in the study.
**Table S4.** RNA concentration, purity and integrity (RIN) of the purified RNA from the indicated strains.

## Data Availability

All datasets generated and analysed during this study are publicly available in the Zenodo repository under the following accession number: DOI 10.5281/zenodo.10696946 (https://zenodo.org/records/10812278).
