# Supplementary material for: Biostimulants in plant brassinosteroid hormone receptor BRI1 activation—a new system to evaluate activation capacity
Source: FEBS J. 2025 Aug 28;293(3):677–97. doi: 10.1111/febs.70235 (PMC12871906; doi:10.1111/febs.70235)
Supplement: Supplementary file 1 — Fig. S1. Plasmid maps of BRI1:GFP and BAK1:mCherry receptors integrated in S. pombe cells. Fig. S2. Plasmid maps of BRI1:3HA and BAK1:13Myc gene receptors (A) and reporters str3:mNG and wwm3:mNG (B). Fig. S3. Expression of BRI1:3HA and BAK1:13Myc gene receptors and activation of str3:mNG and wwm3:mNG reporters by BL. Fig. S4. Activation of str3:mNG and wwm3:mNG reporters by HBPAL, E600 and AA22 biostimulants. Table S1. List of plasmids used in the study. Table S2. List of primers used in the study. Table S3. List of S. pombe strains used in the study. Table S4. RNA concentration, purity and integrity (RIN) of the purified RNA from the indicated strains. [file FEBS-293-677-s001.pdf]

Supplementary Material

Table S1. List of plasmids used in the study.

Plasmid ID	Plasmid name	Source
pAV0751	p*Ura4*^{AfeI}-p^{nmt1}-*sfGFP-terminator*^{ScCYC1}	NBRP/YGRC
pAV0607	p*Ade6*^{PmeI}-p^{act1}-*mCherry-RitC-terminator*^{ScADH1}	NBRP/YGRC
pAV0356	p*Ade6*^{PmeI}	NBRP/YGRC
pAV0751.1	pUra4^{AfeI}-p^{nmt1}-*mCherry-RitC-terminator*^{ScADH1}	This work
pAV0751-*bri1:GFP*	p*Ura4*^{AfeI}-p^{nmt1}-*bri1:sfGFP-terminator*^{ScCYC1}	This work
pAV0751.1-*bak1:mCherry*	p*Ura4*^{AfeI}-p^{nmt1}-*bak1:mCherry-RitC-terminator*^{ScADH1}	This work
pAV0356-*bak1-mCherry*	p*Ade6*^{PmeI}-p^{nmt1}-*bak1:mCherry-RitC-terminator*^{ScADH1}	This work
pAV0751-*bri1:3HA*	p*Ura4*^{AfeI}-p^{nmt1}-bri1:3HA-terminator^{ScCYC1}	This work
pAV0356-*bak1:13Myc*	pAde6^{PmeI}-p^{nmt1}-*bak1:13Myc-RitC-terminator*^{ScADH1}	This work
p*str3:mNG*	p*Leu1*^{XhoI}-*str3:mNeonGreen*	This work
p*wwm3:mNG*	p*Leu1*^{XhoI}-*wwm3:mNeonGreen*	This work

Table S2. List of primers used in the study.

Primer name	Sequence 5'-3'	Use
bak1-Xho1-Fwr	GCGCGCCTCGAGATGGAACGAAGATTAATGATCCCTT GCTTCTTTTGG	*Cloning bak1 into pAV0751.1*
bak1-EcoRI-Rev	CCACTTCCCAGATCGAAAACGAATACCCCTCGGGTCCA AGA GAATTCCGCGCGCG	*Cloning bak1 into pAV0751.1*
cdc2-RT-Fwr	ATTGTCAGGGCGTATTGTGG	qPCR
cdc2-RT-Rev	CACGAATAGCTGTGCTAGGAAC	qPCR
vps66-RT-Fwr	GGTCGTGTTTCTTTAGCCCTTC	qPCR
vps66-RT-Rev	GCCTTTGCAGCAACTTTAGC	qPCR
SPBC1348.07-RT-Fwr	TAAACTTCGTTGGCCCTTGC	qPCR
SPBC1348.07-RT-Rev	ATAGGCCCCCAAATTTCACTG	qPCR
vid21-RT-Fwr	TTGGTTGCCCGAAGAAGATG	qPCR
vid21-RT-Rev	AAAACAATCCCACGCAGTCC	qPCR
str3-RT-Fwr	ATCATGCCAAGCTTCTGTGC	qPCR
str3-RT-Rev	AAATTGGAGACGCGATTGCC	qPCR
wwm3-RT-Fwr	TCCAGGCTTGCATATTCCTC	qPCR
wwm3-RT-Rev	AGCTCCAACCATGAAACCTC	qPCR
edc3-RT-Fwr	AGACCCTGCAAAGCTTCTTG	qPCR
edc3-RT-Rev	TGTGAACCAGCTGAAGGAAG	qPCR
fah1-RT-Fwr	CGTATCTTTAGTCGGCAAGCC	qPCR
fah1-RT-Rev	TGTTTGTGGCGCCATCATAG	qPCR
mug62-RT-Fwr	CGTGTCGCATTTACTACAGAGG	qPCR
mug62-RT-Rev	TGATTTCCACCATGCAAGGC	qPCR
plb4-RT-Fwr	TTCAACGCTCTTTGGAACGC	qPCR
plb4-RT-Rev	TCGCAGCAGACATTACGGTAG	qPCR
SPCC1450.15-RT-Fwr	TTCCTAGCACCATCCTTTCTCC	qPCR
SPCC1450.15-RT-Rev	TTCCTCGCAAGTTTGTACGG	qPCR
sed5-RT-Fwr	GCATGTGTCACGAAAACTCG	qPCR
sed5-RT-Rev	TTGGTGCTTGGTTTGATCGG	qPCR
SPAC607.02c-RT-Fwr	GAGAAGCGTGAAAGAGACTGG	qPCR
SPAC607.02c-RT-Rev	TGTAAATTAGCCCACGGATCAGG	qPCR
SPBC18E5.10-RT-Fwr	AATCCGAATTGAGAGGCAGAGG	qPCR
SPBC18E5.10-RT-Rev	CAGCATTCACAATGACCACTGG	qPCR
nup97-RT-Fwr	CTGACGATTCACCAAAGGAAGC	qPCR
nup97-RT-Rev	TGCGCGTTGTTCAATCTCAC	qPCR
gos1-RT-Fwr	CTGCTAGAGGAAAGAAGGAAGC	qPCR
gos1-RT-Rev	AGTAGCCATTGAATGCGAGTC	qPCR
stc1-RT-Fwr	ACGAAGAGCGAACTGAAAGC	qPCR
stc1-RT-Rev	TCCATCCTGTCTTCATCAGAGTC	qPCR
mug96-RT-Fwr	CCAATTTGCTGCTTTGAGAATGC	qPCR
mug96-RT-Rev	TGGACACTTACCTGACCACAAG	qPCR

Table S3. List of *S. pombe* strains used in the study.

Strain	Genotype	Source/Reference
JM1058	h^+ *his7-366 leu1-32 ade6-m210 ura4-d18*	Lab stock
RA4639	h^+ *his7-366 leu1-32 ade6-m210 ura4-d18 Ura4^{AfeI}-p^{nmt1}-bri1:sfGFP-terminator^{ScCYC1}*	This work
RA4660	h^+ *his7-366 leu1-32 ura4-d18 ade6-m210 Ade6^{PmeI}-p^{nmt1}-bak1:mCherry-RitC-terminator^{ScADH1}*	This work
RA4662	h^+ *his7-366 leu1-32 ura4-d18 ade6-m210 Ura4^{AfeI}-p^{nmt1}-bri1:sfGFP-terminator^{ScCYC1} Ade6^{PmeI}-p^{nmt1}-bak1:mCherry-RitC-terminator^{ScADH1}*	This work
RA4719	h^+ *his7-366 leu1-32 ade6-m210 ura4-d18 Ura4^{AfeI}-p^{nmt1}-bri1:3HA-terminator^{ScCYC1}*	This work
RA4723	h^+ *his7-366 leu1-32 ura4-d18 ade6-m210 Ade6^{PmeI}-p^{nmt1}-bak1:13myc-RitC-terminator^{ScADH1}*	This work
RA4749	h^+ *his7-366 leu1-32 ura4-d18 ade6-m210 Ura4^{AfeI}-p^{nmt1}-bri1:3HA-terminator^{ScCYC1} Ade6^{PmeI}-p^{nmt1}-bak1:13Myc-RitC-terminator^{ScADH1}*	This work
RA4753	h^+ *his7-366 ura4-d18 ade6-m210 leu1-32 Ura4^{AfeI}-p^{nmt1}-bri1:3HA-terminator^{ScCYC1} Ade6^{PmeI}-p^{nmt1}-bak1:13Myc-RitC-terminator^{ScADH1} Leu1^{XhoI}-str3-mNeonGreen*	This work
RA47	h^+ *his7-366 ura4-d18 ade6-m210 leu1-32 Ura4^{AfeI}-p^{nmt1}-bri1:3HA-terminator^{ScCYC1} Ade6^{PmeI}-p^{nmt1}-bak1:13Myc-RitC-terminator^{ScADH1} Leu1^{XhoI}-wwm3-mNeonGreen*	This work

Table S4. RNA concentration, purity, and integrity (RIN) of the purified RNA from the indicated strains (1, 2, 3 represent the replicates for each strain).

Name	Concentration (ng/ul)	Nanodrop 260/280	Nanodrop 260/230	Available (ng)	Quality score (RIN)
WT1	782.90	2.19	1.12	18006.70	9.3
WT2	1323.90	2.19	2.22	30449.70	10.0
WT3	1063.90	2.18	2.23	24469.70	9.8
WTBL1	1029.30	2.17	2.34	23673.90	10.0
WTBL2	1208.10	2.17	2.45	27786.30	10.0
WTBL3	1088.10	2.18	1.38	25026.30	9.2
NBB1	1039.40	2.18	2.39	23906.20	8.9
NBB2	1540.00	2.18	1.42	35420.00	10.0
NBB3	1107.00	2.18	2.26	25461.00	10.0
NBBBL1	950.90	2.19	2.36	21870.70	10.0
NBBBL2	1025.60	2.18	2.45	23588.80	10.0
NBBBL3	1032.40	2.18	1.49	23745.20	10.0
IBB1	1162.10	2.18	2.47	26728.30	10.0
IBB2	1042.70	2.17	2.18	23982.10	10.0
IBB3	919.00	2.19	2.20	21137.00	10.0
IBBBL1	1033.90	2.17	2.49	23779.70	9.9
IBBBL2	1081.30	2.17	1.45	24869.90	10.0
IBBBL3	867.00	2.18	2.33	19941.00	10.0

Supplementary Figures

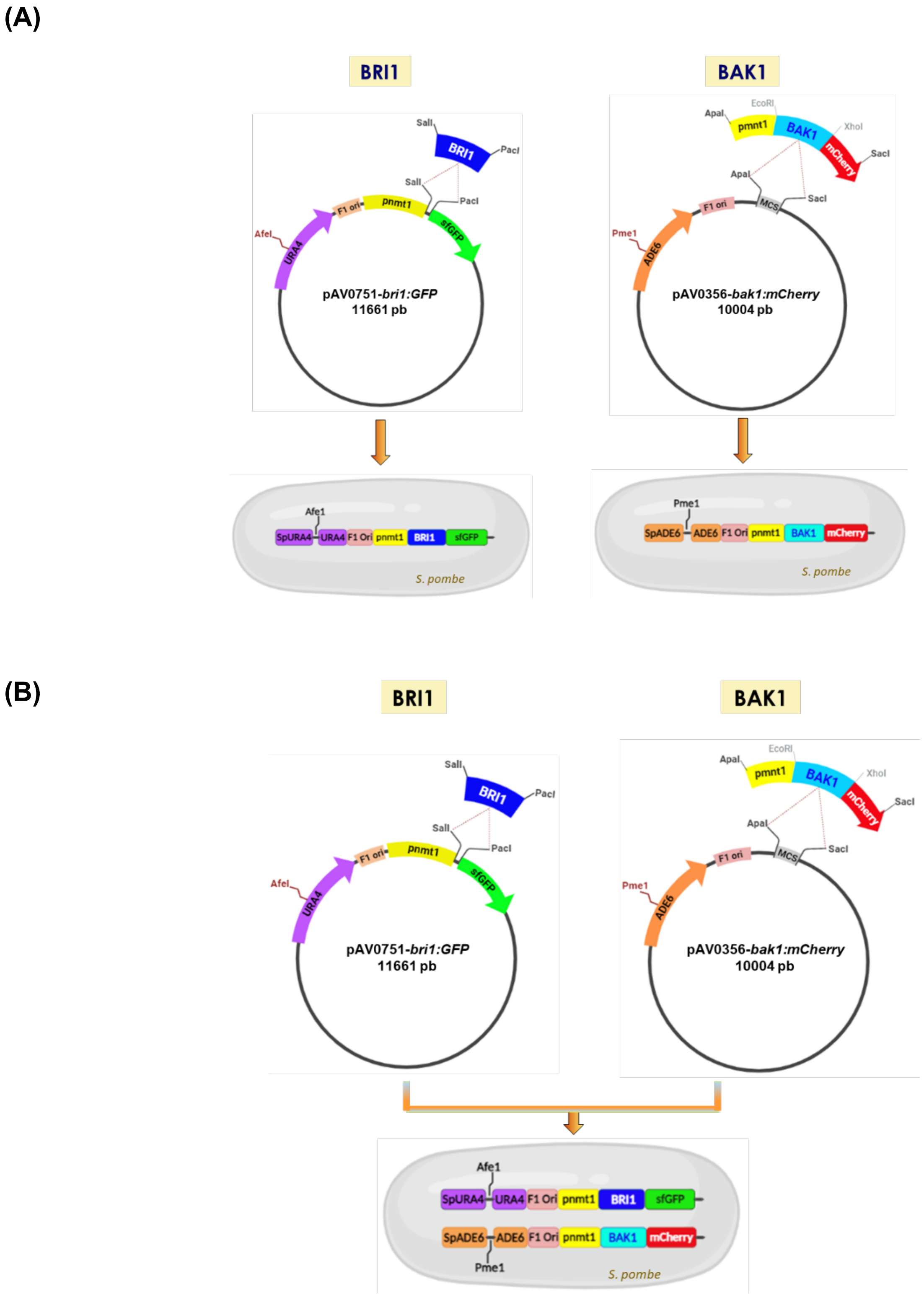

Fig. S1. Plasmid maps of *BRI1:GFP* and *BAK1:mCherry* gene receptors integrated in *S. pombe* cells.

(A)

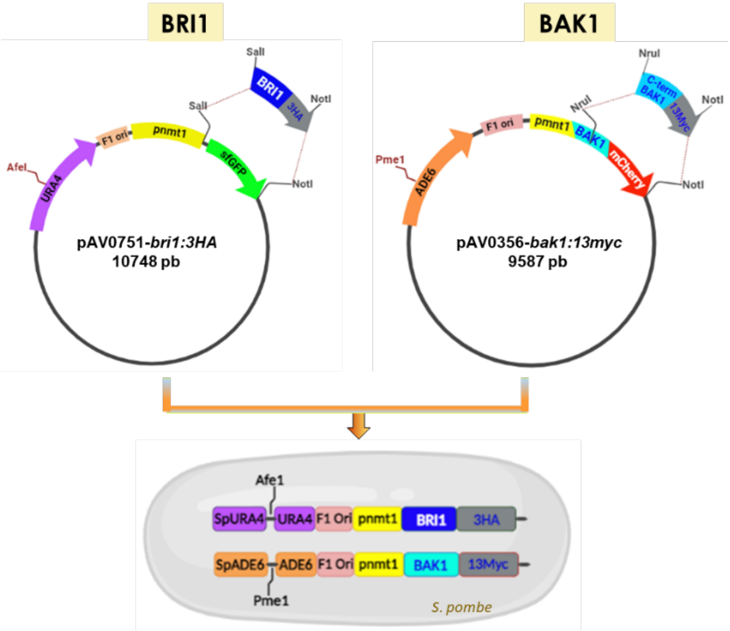

(B)

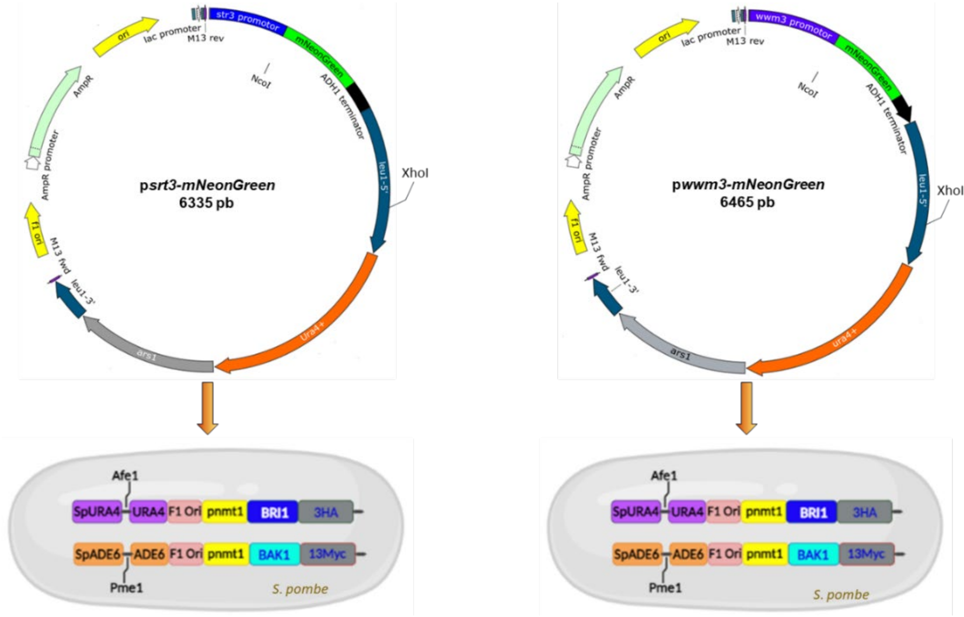

Fig. S2. Plasmid maps of *BRI1:3HA* and *BAK1:13Myc* gene receptors and reporters *str3:mNG* and *wwm3:mNG*. **(A)** Plasmids maps of *BRI1:3HA* and *BAK1:13Myc* gene receptors. **(B)** Plasmid maps of reporters *str3:mNG* and *wwm3:mNG*.

(A)

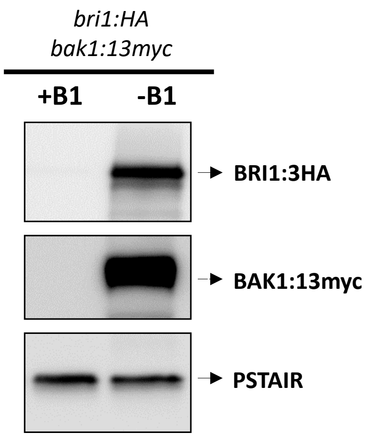

(B)

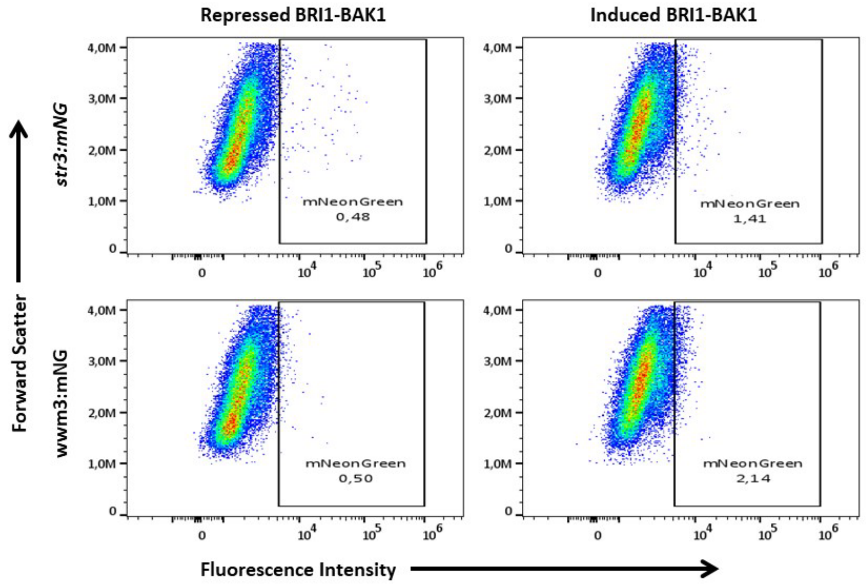

Fig. S3. Expression of BRI1:3HA and BAK1:13Myc receptors and activation of *str3:mNG* and *wwm3:mNG* reporters by BL. (A) Total extracts expressing both BRI1:3HA and BAK1:13Myc proteins through the activation of the inducible *nmt* promoter in the absence of thiamine (-B1) during 17 h or repressing the *nmt* promotor in the presence of thiamine (+B1), were resolved in SD-PAGE and analyzed by western blotting. Blot membranes were cut prior antibodies incubation to detect BRI1:3HA (137 KDa) and BAK1:13Myc (93 KDa) proteins by anti-HA and anti-Myc antibodies respectively. Anti-Cdc2 (PSTAIR) was used as a loading control. **(B)** Detection of *str3-mNG* (upper panels) and *wwm3-mNG* (lower panels) expression by flow cytometry analysis. After repressing (left) or inducing (right) the expression of BRI1-BAK1 receptors for 19 hours, cells were treated with BL 0.4 mM for 3 hours. Frequencies (percentages) of positive cells are shown.

(A)

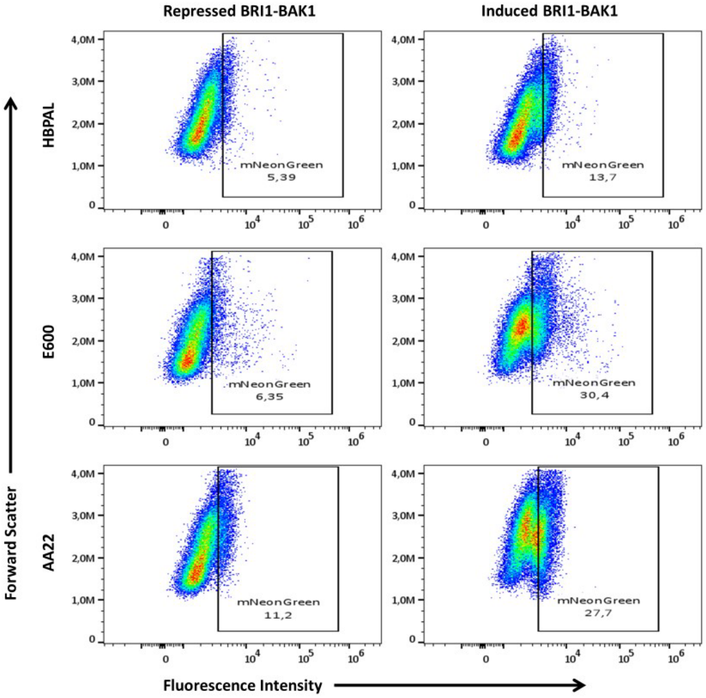

(B)

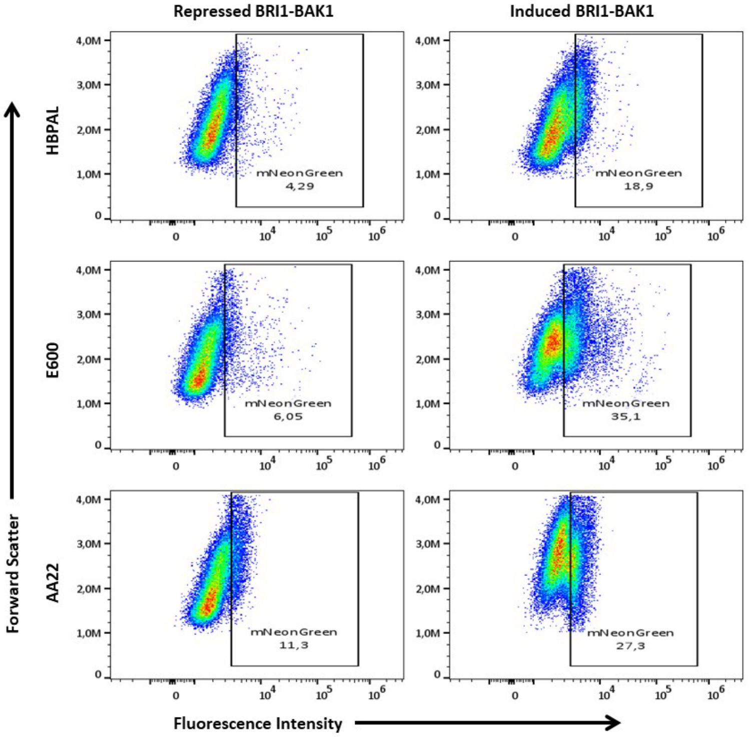

Fig. S4. A**ctivation of *srt3:mNG* and *wwm3:mNG* reporters by HBPAL, E600 and AA22 biostimulants.** Detection of **(A)** *str3-mNG* and **(B)** *wwm3-mNG* expression by flow cytometry analysis. After repressing (left) or inducing (right) the expression of BRI1-BAK1 receptors for 19 hours, cells were treated with HBPAL at 2 µg/µL, E600 at 5 µg/µL or AA22 at 5 µg/µL for 3 hours. Frequencies (percentages) of positive cells are shown.
